# A practical solution to ‘Continuing Care Site’ issues in neonatal clinical trials – a pragmatic approach to regulatory and research governance review

**DOI:** 10.1186/1745-6215-12-S1-A37

**Published:** 2011-12-13

**Authors:** Kayleigh Morgan, Ed Juszczak, Ursula Bowler

**Affiliations:** 1 NPEU Clinical Trials Unit, University of Oxford, Oxford, UK

## 

I2S2 is a randomised controlled trial of iodine supplementation in preterm infants examining whether iodine supplementation can improve neurodevelopmental outcome at two years of age. 20 Neonatal Units in the UK will recruit around 1400 infants <31 weeks of gestation into the trial; infants will receive a daily dose of sodium iodide or sodium chloride placebo until 34 week’s corrected age.

Due to the nature of neonatal care, approximately 50% of infants participating in I2S2 are likely to be transferred from the ‘Recruiting Site’ to another hospital for continuation of their clinical care. Hospitals which are not the primary research site may receive infants with little warning and be required to continue treatment under the trial protocol. Hospitals which are research active but have a reduced level of involvement have been defined as ‘Continuing Care Sites’ and ‘Data Collection Sites’; such sites require Research Management and Governance review proportionate to their level of involvement in the clinical trial.

This three-tiered approach was negotiated between the NPEU Clinical Trials Unit, R&D Forum and the National Institute for Health Research Clinical Research Network Coordinating Centre (NIHR CRN CC), with contributions from and supported by the Birmingham and Black Country Comprehensive Local Research Network (BBC CLRN) and the University of Oxford. It was approved by the Medicines and Healthcare products Regulatory Agency and Research Ethics Service as part of a substantial amendment. The use of a generic Site-Specific Information (SSI) and supporting document entitled ‘Statement of Responsibilities’ clearly defines site involvement, NHS permission and funding; both documents negate the need for site agreements with the sponsor. NHS permissions are granted for each Neonatal Unit across the UK; those sites without identified Principal Investigators issue provisional NHS Trust approval with confirmation of Principal Investigator on point of transfer. Appropriate trial specific training materials are provided to all sites including training in the reporting of SAE’s and SUSAR’s.

This approach allows NHS permissions to be issued in advance of any transfer and ensures infants recruited to I2S2 can continue trial procedures at different sites. Withdrawing an infant from the trial because regulatory and research governance paperwork is not in place, seems a wasted effort for all individuals involved, and more importantly unethical to parents. This streamlined, pragmatic approach to governance review has proved a successful strategy from which many can learn.

Trial registry [http://www.clinicaltrials.gov]; Identifier: NCT00638092. Funded by UK Medical Research Council.

